# Rapid Extraction and Detection of African Swine Fever Virus DNA Based on Isothermal Recombinase Polymerase Amplification Assay

**DOI:** 10.3390/v13091731

**Published:** 2021-08-31

**Authors:** Arianna Ceruti, Rea Maja Kobialka, Judah Ssekitoleko, Julius Boniface Okuni, Sandra Blome, Ahmed Abd El Wahed, Uwe Truyen

**Affiliations:** 1 Institute of Animal Hygiene and Veterinary Public Health, Leipzig University, 04103 Leipzig, Germany; arianna.ceruti@uni-leipzig.de (A.C.); rea_maja.kobialka@uni-leipzig.de (R.M.K.); truyen@vetmed.uni-leipzig.de (U.T.); 2 College of Veterinary Medicine, Animal Resources and Biosecurity (COVAB), Makerere University, Kampala P.O. Box 7062, Uganda; jsekitoleko2810@gmail.com (J.S.); jbokuni@gmail.com (J.B.O.); 3 National Agricultural Research Organisation, Entebbe P.O. Box 295, Uganda; 4 Friedrich-Loeffler-Institut, Institute of Diagnostic Virology, 17493 Greifswald, Germany; Sandra.Blome@fli.de

**Keywords:** African swine fever virus, recombinase polymerase amplification, DNA extraction, molecular detection

## Abstract

African swine fever virus (ASFV) is the causative agent of a deadly disease in pigs and is spread rapidly across borders. Samples collected from suspected cases must be sent to the reference laboratory for diagnosis using polymerase chain reaction (PCR). In this study, we aimed to develop a simple DNA isolation step and real-time recombinase polymerase amplification (RPA) assay for rapid detection of ASFV. RPA assay based on the p72 encoding B646L gene of ASFV was established. The assays limit of detection and cross-reactivity were investigated. Diagnostic performance was examined using 73 blood and serum samples. Two extraction approaches were tested: silica-column-based extraction method and simple non-purification DNA isolation (lysis buffer and heating, 70 °C for 20 min). All results were compared with well-established real-time PCR. In a field deployment during a disease outbreak event in Uganda, 20 whole blood samples were tested. The assay’s analytical sensitivity was 3.5 DNA copies of molecular standard per µL as determined by probit analysis on eight independent assay runs. The ASFV RPA assay only detected ASFV genotypes. Compared to real-time PCR, RPA diagnostic sensitivity and specificity were 100%. Using the heating/lysis buffer extraction procedure, ASFV-RPA revealed better tolerance to inhibitors than real-time PCR (97% and 38% positivity rate, respectively). In Uganda, infected animals were identified before the appearance of fever. The ASFV-RPA assay is shown to be as sensitive and specific as real-time PCR. Moreover, the combination of the simple extraction protocol allows its use at the point of need to improve control measures.

## 1. Introduction

African swine fever causes a highly lethal, contagious disease in pigs, threatening the global swine industry and national economies. Accordingly, the virus is placed on the list of notifiable diseases of the World Organization for Animal Health (OIE).

The virus is a member of the *Asfarviridae* family [1] with an envelope and a large double-stranded DNA genome of 170–193 kbp [2]. In total, 24 genotypes and 8 serotypes were discovered mainly in Africa, [3,4,5,6,7,8], where ASFV was first described in Kenya in 1921 [9]. The virus is circulating in a sylvatic cycle among African wild suids (mainly Warthogs, *Phacochoerus africanus*) and *Ornithodoros* soft ticks in sub-Saharan Africa. This cycle is not accompanied by overt disease [10]. Globally, genotypes I and II are the major causes of outbreaks with direct transmission between wild and domestic pigs. Since its first Introduction into Portugal in 1957 [11], the virus had been circulating through southern European countries until the late 1990s. Europe has faced the remerging of ASFV in Georgia in 2007 [12]. Recently, many cases have been identified in wild pigs in Germany and Poland [13,14,15,16]. A key aspect that facilitates its widespread transmission is the various transmission modes: arthropod vector (sylvatic cycle) mainly in Africa, direct or indirect contact with contaminated secretions (of either wild boars, Sus scrofa, or domestic pigs), as well as inanimate fomites (e.g., clothes, transport vehicles, carcasses, contaminated pork) [17].

Clinical signs associated with ASF are highly variable, ranging from peracute (lethality 90–100%) to asymptomatic, depending on various factors, e.g., the virulence of the virus, viral infectious dose, and host genetic background [18]. The most common form is the acute infection that induces high fever, lethargy, respiratory and digestive dysfunctions (often with hemorrhagic tendency), abortion, and sudden deaths. Since it shows great similarities with other infectious diseases regarding clinical and pathological pictures (e.g., classical swine fever (CSF)) [19], differential laboratory diagnosis is essential. The host range of ASFV is restricted to swine and no records of other livestock or human infection have been reported. Since neither effective treatment nor vaccination are available, the most essential control measures are identification of infected animals in wild or domestic pigs and immediate culling and movement restriction.

African swine fever virus can be isolated on macrophage cultures or on bone marrow cells, which requires a highly equipped laboratory. There are recommended direct (antigen or whole virus) and indirect (antibody) detection methods for ASFV. Indirect techniques comprise serological assays based on antibody enzyme-linked immunosorbent assays (ELISA), indirect immunoperoxidase test, and immunoblotting. Direct methods include hemadsorption test, virus isolation on macrophages, antigen detection by fluorescent antibody test, or antigen ELISA. The gold standard, however, is molecular genome detection based on polymerase chain reaction (PCR), either conventional or real-time. Many PCR assays were established over the past 20 years and recommended by the OIE [20,21,22], but PCR testing is limited to regional or reference laboratories, because of the complexity of the PCR and for biosafety reasons. A simpler and more standardized approach has been shown to be useful in less equipped laboratories [23]. Moreover, an on-site detection system will save time and decrease the duration between sample collection and results, which lead to the immediate implementation of control measures. Recently, promising isothermal amplification methods were developed and used to detect other animal pathogens [24,25]. Nevertheless, a key aspect that makes the implementation of molecular point-of-care tests still challenging is the lack of simple and effective on-site nucleic acid extraction. Among rapid molecular assays is recombinase-based isothermal amplification: Recombinase polymerase amplification (RPA) and recombinase-aided amplification (RAA). The chemical process relies on three core enzymes and proteins: a recombinase (uvsX of T4 phage for RPA or the recombinant enzyme from E. coli for RAA), single-stranded DNA-binding protein (SSB), and the polymerase. These chemicals replace the denaturation, annealing, and extension steps of the PCR, but at a constant temperature of 37–42 °C for a maximum of 15 min. Furthermore, the detection of real-time amplification is based on a synthetic molecular probe [26], which emits fluorescence upon binding to the amplicon.

In this study, a rapid DNA extraction and RPA assay targeting the B646L gene (encoding the capsid protein p72) of ASFV was developed. The limit of detection, cross reactivity, and clinical performance were also determined. All results were compared with a reference silica gel-column-based extraction method and real-time PCR.

## 2. Materials and Methods

### 2.1. Clinical Samples and Ethical Statement

In total, 52 whole blood samples from experimentally infected domestic pigs were used in the study. The animal experiment was externally approved by the competent authority (Landesamt für Landwirtschaft, Lebensmittelsicherheit und Fischerei (LALLF) Mecklenburg-Vorpommern) under reference number 7221.3-2-011/19. In addition, 21 serum samples from routine diagnosis submitted to the faculty of Veterinary Medicine, Leipzig University, Germany, were screened.

### 2.2. Molecular DNA Standard and RPA Oligonucleotides

The B604L gene was used as the target for the developed RPA assay. A 417-nucleotide-long molecular standard (GenBank accession number: MK554698.1; nt: 1491-1908) was synthesized by Thermo Fisher Scientific GENEART (Regensburg, Germany). The RPA oligonucleotides were selected based on multiple alignment of 24 sequences representing the ASFV genotypes (Accession Numbers: AF302816, AM999764, AF270706, FJ528594, DQ250120, AF302818, AY494553, AF270711, AF302818, AF270705, AY351564, AF449463, AY351522, AY351543, AY351542, AY351555, AY494552, AY494551, DQ250119, DQ250122, DQ250127, DQ250109, DQ250125, DQ250117, KT795360, KY353989) using Geneious 2020.2.3 (https://www.geneious.com, accessed on 2 October 2020). Six primers and one exo-probe were designed and screened in this study (Table 1). The primers/probe combination yielding the highest signal in RPA (threshold time (TT) in minutes and fluorescence intensity in millivolt (mV)) was selected for further assay validation. TIB MOLBIOL GmbH (Berlin, Germany) synthesized all oligonucleotides.

### 2.3. RPA Conditions

A real-time RPA assay was performed in a 50 µL volume using the TwistAmp Exo kit (TwistDx, Cambridge, UK). The reaction mix comprised 29.5 µL rehydration Buffer, 8.2 µL nuclease-free water, 2.5 µL magnesium acetate (280 mM), 2.1 µL of each primer (10 µM), 0.6 µL probe (10 µM), and 5 µL template (or 1 µL for samples treated with the rapid extraction protocol), which was added into the lid of the reaction tube containing the freeze-dried pellet. Water was used instead of the DNA template for the negative control. For RNA viruses tested for cross reactivity, 8.2 µL (500 U) of RevertAid reverse transcriptase (Thermo Scientific, Regensburg, Germany) was used instead of nuclease-free water. The tube was closed, centrifuged, mixed, centrifuged, and placed immediately into the T8-ISO Instrument (Axxin, Fairfield, Australia). The incubation temperature was 42 °C for 15 min. A mixing and centrifuging step was conducted at 320 s after the test start. The FAM fluorescence signal was recorded in real time. The TT was determined using the T8 Desktop Application (version 2.8.0.0, Axxin) based on the first derivative values.

### 2.4. Analytical Sensitivity and Specificity

To determine the real-time ASFV-RPA assay’s analytical sensitivity, eight replicates of serial dilutions of the molecular standard (10^2^-10^0^ DNA Copies per µL) were tested. The limit of detection was calculated using RStudio version 1.3.1093 [27] performing a probit regression analysis and visualized using the ggplot2 package (v3.3.3) [28]. Cross reactivity of the real-time RPA assay was determined using nucleic acids of viruses listed in Table 2.

### 2.5. Nucleic Acid Extraction Procedures

DNAs from samples were extracted by two different methods. First, a standardized silica-based DNA extraction kit (DNAeasy Blood & Tissue Kit, QIAGEN GmbH, Hilden, Germany) was used for the purification of total DNA, as instructed by the manufacturer. A total of 5 µL was used as a template in the RPA reaction. Second, the same clinical samples were incubated with 200 µL QIAGEN ATL lysis buffer at 70 °C for 20 min. Then, 1 µL of the processed sample was diluted in 9 µL nuclease-free water, and 1 µL of the mix was used as a template.

### 2.6. Real-Time PCR Conditions

The molecular standard as well as all clinical samples were tested with an established real-time PCR targeting the same gene region of the ASFV-RPA assay [20]. The real-time PCR was performed on the Stratagene M × 3000 P QPCR from Agilent Technologies (Santa Clara, California, United States). The reaction of QuantiNova Probe PCR kit (QIAGEN GmbH, Hilden, German) consisting of 12.5 µL of the QuantiNova Probe PCR Master Mix, 5 µL H_2_O, 1 µL of each primer (10 µM), 0.5 µL of probe (10 µM), and 5 µL template, reaching a total volume of 25 µL. The following temperature profile was used: 95 °C for 2 min for initial denaturation; 40 cycles of amplification including 10 s at 94 °C and 30 s at 60 °C for denaturation and annealing, respectively.

### 2.7. Pilot Field Deployment

On request of a small farm in Kibaale district in Uganda, 20 whole blood samples of suspected ASF domestic pigs were screened at Makerere University (Kampala, Uganda). DNA was extracted using a Quick-gDNATM MiniPrep kit from ZYMO Research (Irvine, CA, United States) according to the manufacturer’s instructions. For the heating/lysis buffer method, samples were incubated with 200 µL genomic lysis buffer from the Miniprep kit at 70 °C for 20 min. RPA was performed as mentioned above.

## 3. Results

### 3.1. Selection of RPA Primers and Probe

All possible primer combinations were tested using a concentration of 10^5^ of the ASF molecular standard. Best results were achieved using FP1 and RP3 with a TT of 2.66 min and a fluorescence signal of 5000 mV (Supplementary Figures S1–S3). This primer combination was used for further assay validation.

### 3.2. Analytical Sensitivity and Specificity

**Figure 1:**
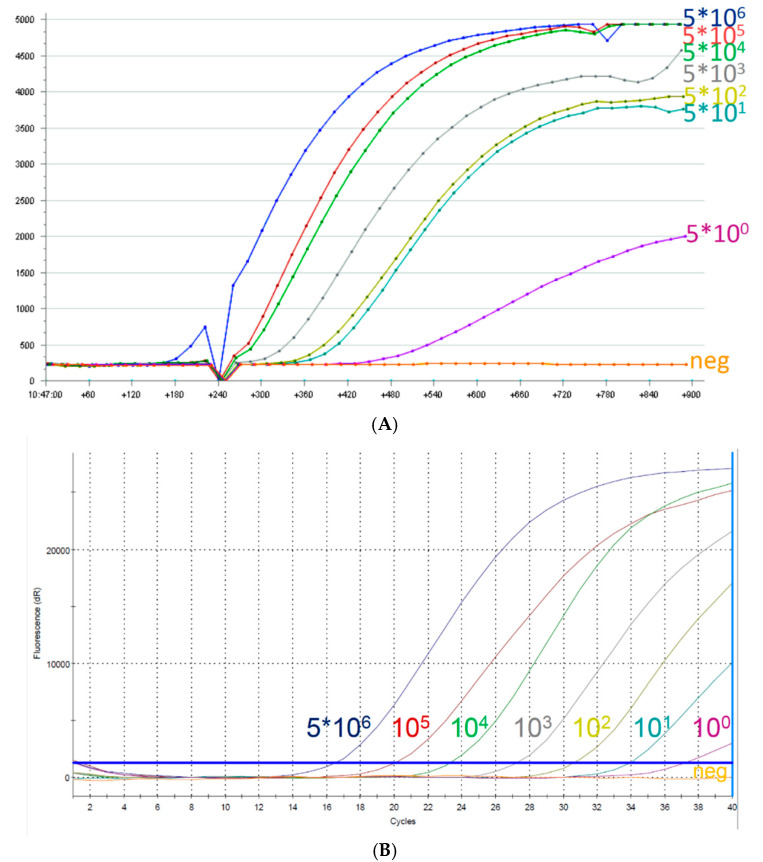
Amplification curves of RPA (**A**) and real-time PCR (**B**). Both assays detected down to one DNA molecule per µL.

To compare the performance of the RPA to the real-time PCR using molecular standard, a serial dilution of 5 * 10^6^–5 * 10° DNA molecules/reaction was prepared and tested. Both assays were able to amplify and detect down to one DNA molecule/µL (Figure 1).

**Figure 2:**
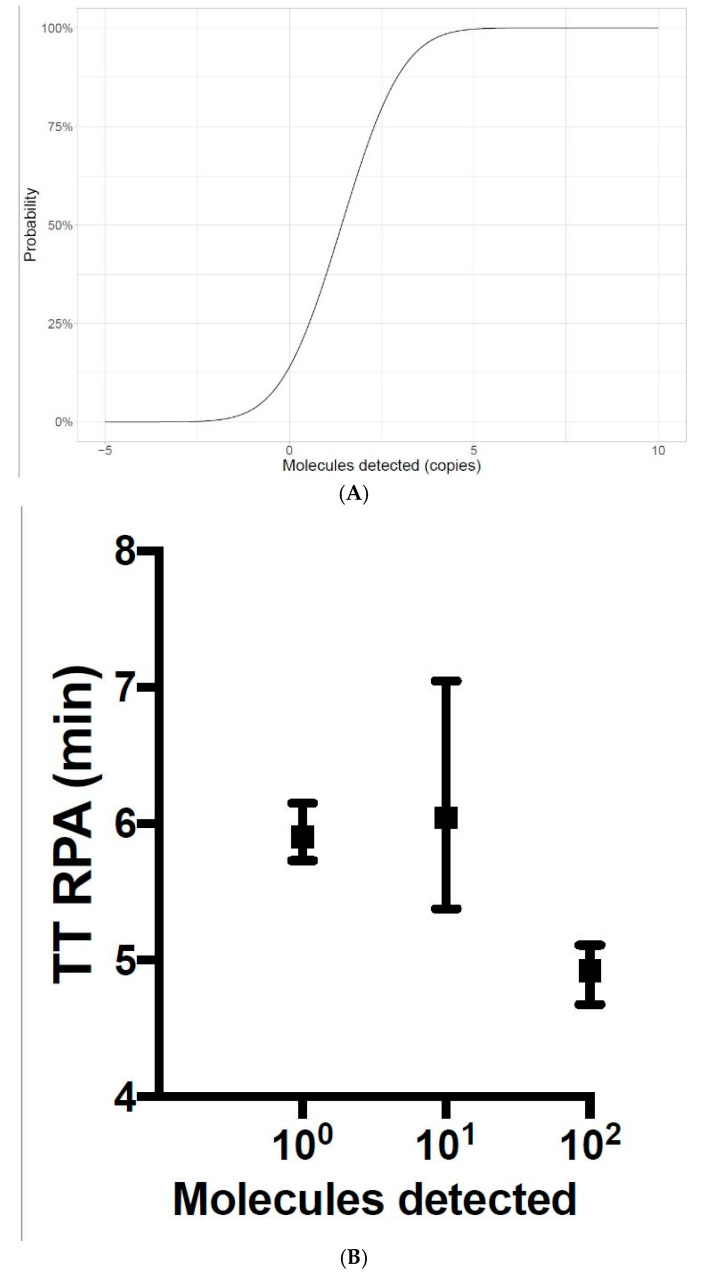
Limit of detection (**A**) and reproducibility (**B**) of the ASF RPA assay. Dataset of 8 RPA runs of the molecular standard 100 to 1 DNA copy/μL was used. Limit of detection is 3.5 copies/µL (A). The speed of the assay was between 5 and 7 min (B).

To determine the RPA assay limit of detection, eight RPA runs of 100, 10 and 1 molecular standard DNA molecules/μL were performed. The 100 and 10 copies/μL were detected in all the 8 runs (8/8 runs), while the 1 copy/μL was identified in 3/8 runs. With this dataset, a probit regression analysis was performed and yielded a limit of detection of 3.5 copies per µL (95% CI) (Figure 2A). The reaction speed was under 7 min (Figure 2B). The ASFV-RPA assay detected all tested ASFV nucleic acids as positive (Table S1) and did not cross-react with nucleic acids of other viruses (Table 2).

### 3.3. Clinical Samples

**Figure 3:**
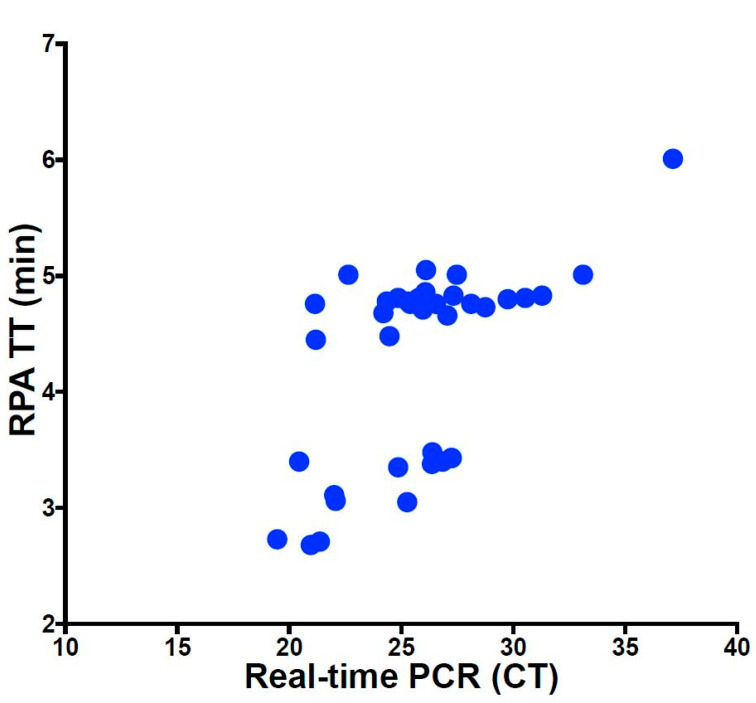
Comparison between the TT value of the RPA and Ct value of the real-time PCR. No correlation was found (R^2^ = 0.34) as the RPA is much faster than the real-time PCR.

DNAs from silica gel extraction protocol and simple heat/lysis step were screened simultaneously in both real-time PCR and RPA assay. By using the pure DNA from the silica-gel-based method, both real-time PCR and RPA have correctly detected 37 as positive and 36 as negatives (Table 3, Table S1 Supplementary file). No correlation between the Ct of the real-time PCR and the TT of the RPA was detected (Figure 3). When applying the simple heat/lysis step, 36 samples were assigned as positive, 1 as a false negative, and 36 as negatives. In contrast, in real-time PCR, only 14 out of the 37 positive samples were detected (Table 3, Supplementary Table S1).

### 3.4. Field Deployment in Low-Resource Settings

**Figure 4:**
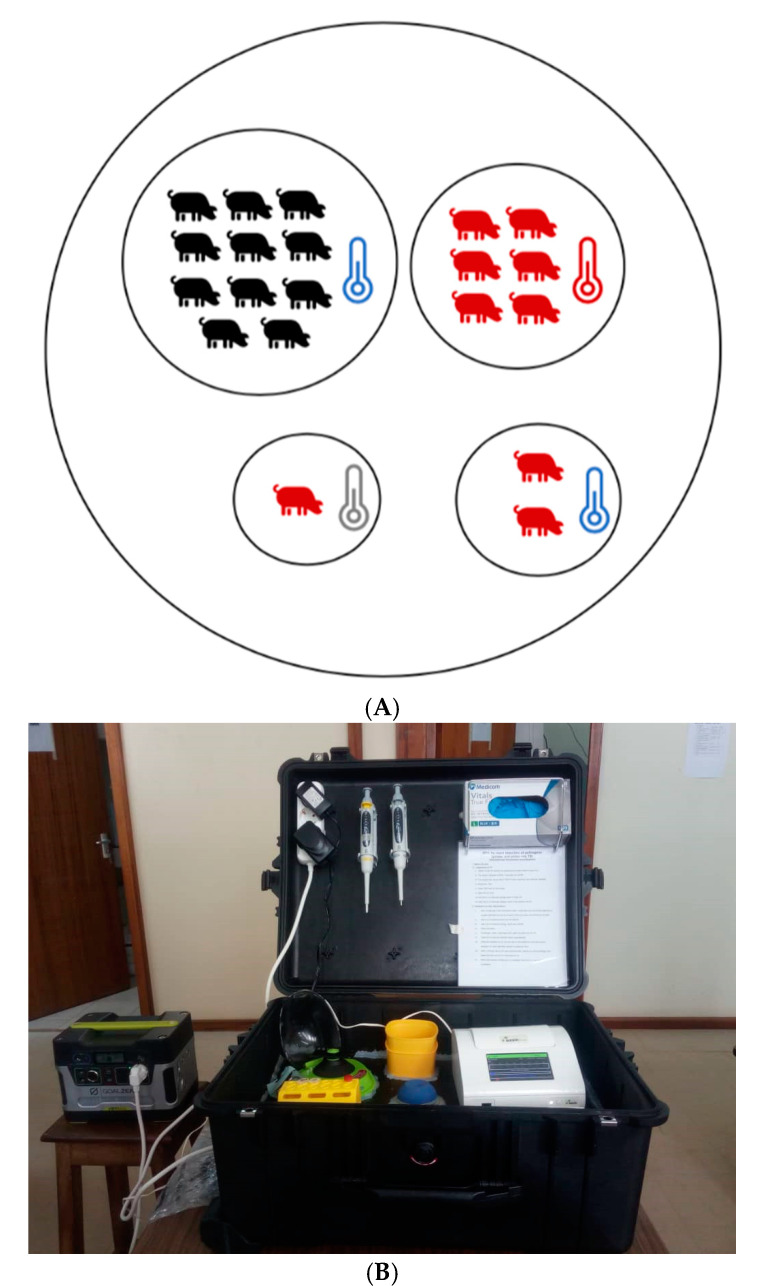
Field deployment of mobile suitcase lab in Uganda: (**A**) ASFV-RPA assay results of 20 samples from suspected ASF domestic pigs in Uganda. Eleven afebrile pigs tested negative, while six febrile pigs, one pig without temperature reading and two afebrile pigs tested positive. Red is animal tested positive. Black is animal tested negative. Blue thermometer indicates normal body temperature, red is pig with fever, and grey is pig with no body temperature measured. (**B**) Mobile suitcase lab.

Blood samples from domestic pigs from an outbreak in Uganda were tested with the ASFV-RPA assay combined with either heating/lysis buffer method or routine silica-gel-based nucleic acid purification method. Eleven samples were assigned as negative and nine as positive using both extraction methods (Figure 4). TT values between the two extraction methods did not differ considerably. Using the silica-based extracted DNA, TT values were between 3.1 and 5.45 min; using the rapidly extracted DNAs between 5.11 and 6.05 min.

## 4. Discussion

ASFV detection relies on well-equipped reference laboratories performing established diagnostic methods. In the present study, we developed a sensitive and specific real-time RPA assay for the rapid detection of ASFV. The B646L gene, encoding for the major capsid protein p72, was chosen as a target since it is a highly conserved region [29,30]. The ASFV-RPA assay was as sensitive as the OIE-recommended real-time PCR being able to detect down to one DNA copy/μL. Moreover, no cross reaction was observed with other viruses with a similar clinical picture. The bottleneck of molecular point-of-need testing remains sample inactivation and extraction. Therefore, the ASFV-RPA assay was also combined with a simple heating and lysis buffer procedure for blood samples, showing a 97% positivity rate.

The gold standard detection method is real-time PCR, which takes up to several hours to deliver results. Two modified PCR assays have been developed to speed up the testing and simplifying the extraction method [31,32]. The total run time was two hours for nine samples implementing a cartridge-based kit, which is easily deployable but required various hands-on steps per sample [32]. When using a magnetic-bead-based extraction protocol, an automated expensive device was required [31]. Direct use of blood in PCR inhibited the reaction as observed in our study (sensitivity 38%) and by others [31,33]. Around 5 to 10 shifts in the Ct values were observed in our study when comparing highly purified DNA and non-processed blood samples. In contrast, RPA is better suited for crude blood samples without further purification steps (sensitivity 97%). No differences were recorded in the TT RPA values between the DNAs of the two extraction approaches. The tolerance of the RPA assay to inhibitors such as milk, hemoglobin, ethanol, and heparin was reported [34,35].

The assay speed is crucial, especially at the point-of-need testing. When comparing the performance of RPA and real-time PCR using linear regression analysis, no correlation was found between TT and Ct values (Figure 3). The reason is the explosive nature of the RPA reaction at a single constant temperature leading to a non-linear amplification outcome [36], while the thermal cycling profiles needed for the PCR reaction lead to more regular exponential amplification curves [37]. Both the speed and robustness of the ASFV-RPA make it an ideal candidate for point-of-need testing. Other advantages are the stability of reagents at ambient temperature (around 40 °C for up to 3 months) and operation in a portable mobile suitcase laboratory [34,38,39,40]. The field study in Uganda showed the successful deployment of the ASFV-RPA assay in low-resource settings. In addition, afebrile animals carrying the virus were detected before the onset of clinical signs. Thus, a deployment for early ASFV screening is viable and can help early control of the disease. Moreover, our study is the first to test clinical ASF samples both from Europe and eastern Africa using a point-of-need setup.

Many isothermal amplification assays have been developed over the past two decades for identifying ASFV. Loop-mediated isothermal amplification (LAMP) [41,42] and cross-priming amplification (CPA) [43,44] detected ASFV with sensitivity of 90 and 70%, respectively [45]. Both required 3-6 sets of primers to amplify the target region, in addition, the run time was around 30–60 min at temperatures >50 °C. The results visualization was based on SYBR Green. In ASFV-RPA assay, five DNA copy was amplified using two primers in less than 10 min and with higher specificity applying an exo-probe-based system.

RPA assays for the detection of ASFV based on separate steps of amplification and visualization using lateral flow were developed [33,46]. The clinical sensitivities of these assays were ranging between 70 and 100% with a turnaround time of 30 min. This approach is subjective to high cross-contamination risk since the post-amplification pipetting step is needed to transfer the amplicon to the lateral flow cartridge. RPA assay relying on CRISPR as a reporter is highly sensitive but has a runtime similar to the real-time PCR and the reagents must be stored at −20 °C [46]. Other ASFV-RPA amplification monitoring based on SYBRE Green dye is not field applicable because of the need to open the post-amplification tube to add the dye [47]. The developed real-time exo-probe based ASFV-RPA assay in this study is highly sensitive, produces faster results (<10 min), and utilizes an all-in-one tube reaction mix. The only drawback is the need for a fluorometer, which adds to the start-up costs. Two other real-time ASFV-RPA and RAA assays have been established but were limited to samples originated from China [48,49] and did not amplify properly our isolate from Germany (Supplementary Figure S4). Therefore, we recommend testing local isolates before implementing diagnostics for ASFV to avoid false negatives.

In conclusion, the developed probe-based real-time RPA assay is shown to be a highly sensitive and specific detection method for ASFV. Furthermore, the simple and effective heating/lysis buffer extraction procedure eases the on-site applicability of the assay. When combining ASFV-RPA with a portable lab setup, e.g., mobile suitcase lab, it can be deployed in the field as point of need testing method. This would allow faster detection of ASF cases since it can significantly reduce the time between sample collection and result.

## Figures and Tables

**Table 1:** RPA oligonucleotides sequences.

ID	Sequence (5′ to 3′)
Probe	ATCGATAAATTTCCATCAAAGTTCTGCAGC-BHQ1-THF-FAM-TACATACCCTTCCAC
FP1	TGGTATCAATCTTATCGATAAATTTCCATCAA
FP2	CCTATTATTAAAAACATTTCCGTAACTGCTCA
FP3	ATATTAGCCCCGTTACGTATCCGATCACATTA
RP1	AATTCTCTTGCTCTGGATACGTTAATATGACC
RP2	ACTGGGTTGGTATTCCTCCCGTGGCTTCAAAG
RP3	CAAAGGTAATCATCATCGCACCCGGATCATCG

**Table 2:** List of viruses whose nucleic acids were included in the cross-specificity testing.

Virus Name	Virus Type	Number of Samples
African swine fever virus	DNA, enveloped, double-stranded	10
Classical swine fever virus	RNA, enveloped, single-stranded	11
Porcine parvovirus (NADL-2)	DNA, non-enveloped, single-stranded	1
Foot and mouth disease virus	RNA, non-enveloped, single-stranded	10
Modified vaccinia Ankara	DNA, enveloped, double-stranded	1
Porcine circovirus-2	DNA, non-enveloped, single-stranded	1

**Table 3:** Sensitivity and specificity of ASFV-RPA and real-time PCR using two extraction procedures. Sensitivity was significantly lost in PCR using rapid heat/lysis extraction with blood samples.

Extraction Method	Sensitivity (*n* = 37)		Specificity (*n* = 36)	
	RPA	Real-Time PCR	RPA	Real-Time PCR
Qiagen DNeasy Blood & Tissue kit	100%	100%	100%	100%
Heated sample in lysis buffer	97%	38%	100%	100%

## Data Availability

All data produced in the study are mentioned in the manuscript or supplementary materials.

## Supplementary Materials

The following are available online at https://www.mdpi.com/article/10.3390/v13091731/s1. Figure S1: Possible primer combinations tested with 5 × 10^5^ ASFV molecular standard; Figure S2: The three best primer combinations tested with 5 × 10^5^ ASFV molecular standard, Figure S3: The two best primer combinations were tested with 5 × 10^3-1^ ASFV molecular standard, Table S1: ASFV positive samples. ASFV Genotype and sample matrix were listed, Figure S4: RPA assay using 10^3-1^ of the ASFV molecular standard based on Wang et al. 2020.
