# Supplementary material for: Rapid Extraction and Detection of African Swine Fever Virus DNA Based on Isothermal Recombinase Polymerase Amplification Assay
Source: Viruses. 2021 Aug 31;13(9):1731. doi: 10.3390/v13091731 (PMC8472937; doi:10.3390/v13091731)
Supplement: Supplementary file 1 [file viruses-13-01731-s001.zip › viruses-1339947-supplementary.pdf]

Supplementary file #1

Rapid extraction and detection of African swine fever virus DNA based on isothermal recombinase polymerase amplification assay

Arianna Ceruti[1]*, Rea Maja Kobialka[1], Judah Ssekitoleko[2], Julius Boniface Okuni[2], Sandra Blome[3], Ahmed Abd El Wahed[1], Uwe Truyen[1]

[1] Institute of Animal Hygiene and Veterinary Public Health, Leipzig University, Germany; rea_maja.kobialka@uni-leipzig.de , ahmed.abd el wahed@uni-leipzig.de, truyen@vetmed.uni-leipzig.de,
[2] College of Veterinary Medicine, Animal Resources and Biosecurity (COVAB), Makerere University, Uganda; jsekitoleko2810@gmail.com, jbokuni@gmail.com
[3] Friedrich-Loeffler-Institut, Institute of Diagnostic Virology, Greifswald, Germany; Sandra.Blome@fli.de
* Correspondence: ahmed.abd_el_wahed@uni-leipzig.de

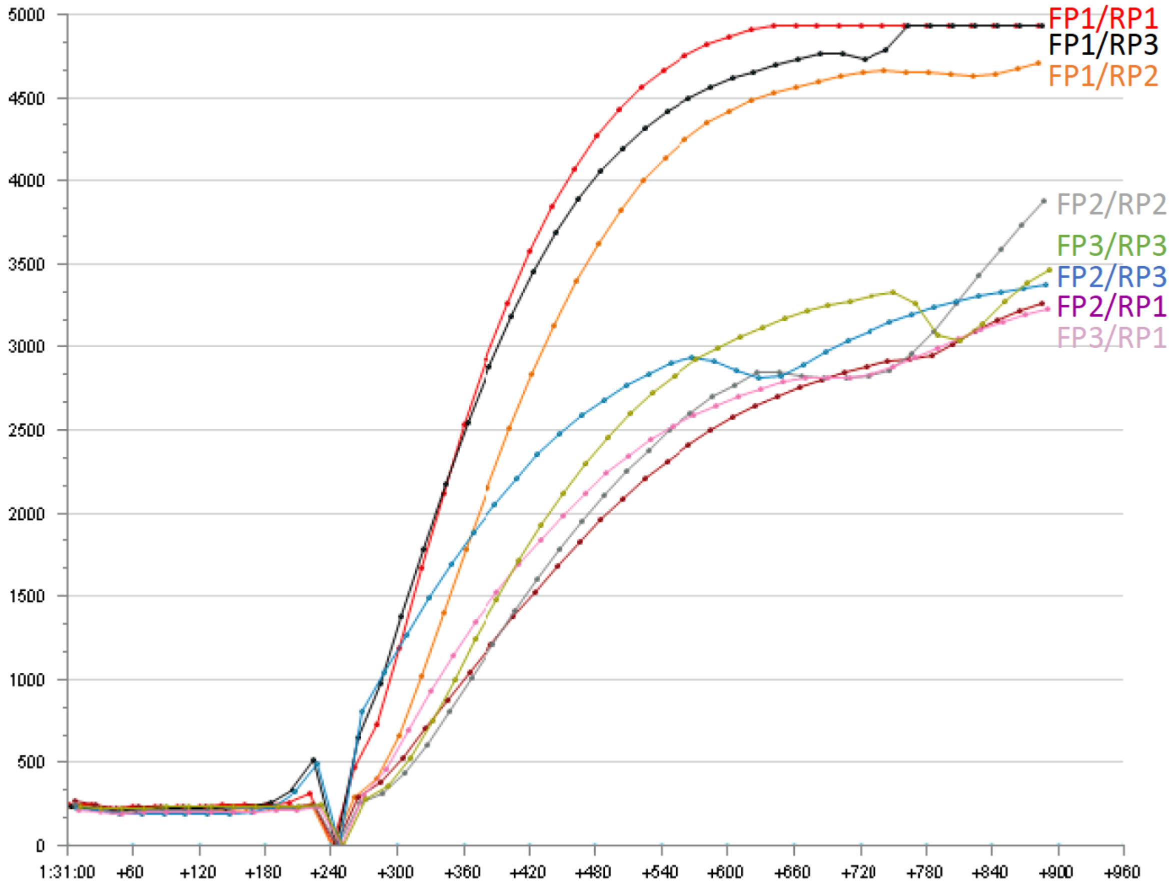

Figure S1. Possible primer combinations tested with 5*10^5 ASFV molecular standard. FP1/RP3 had the fastest TT and maximal fluorescence signal.

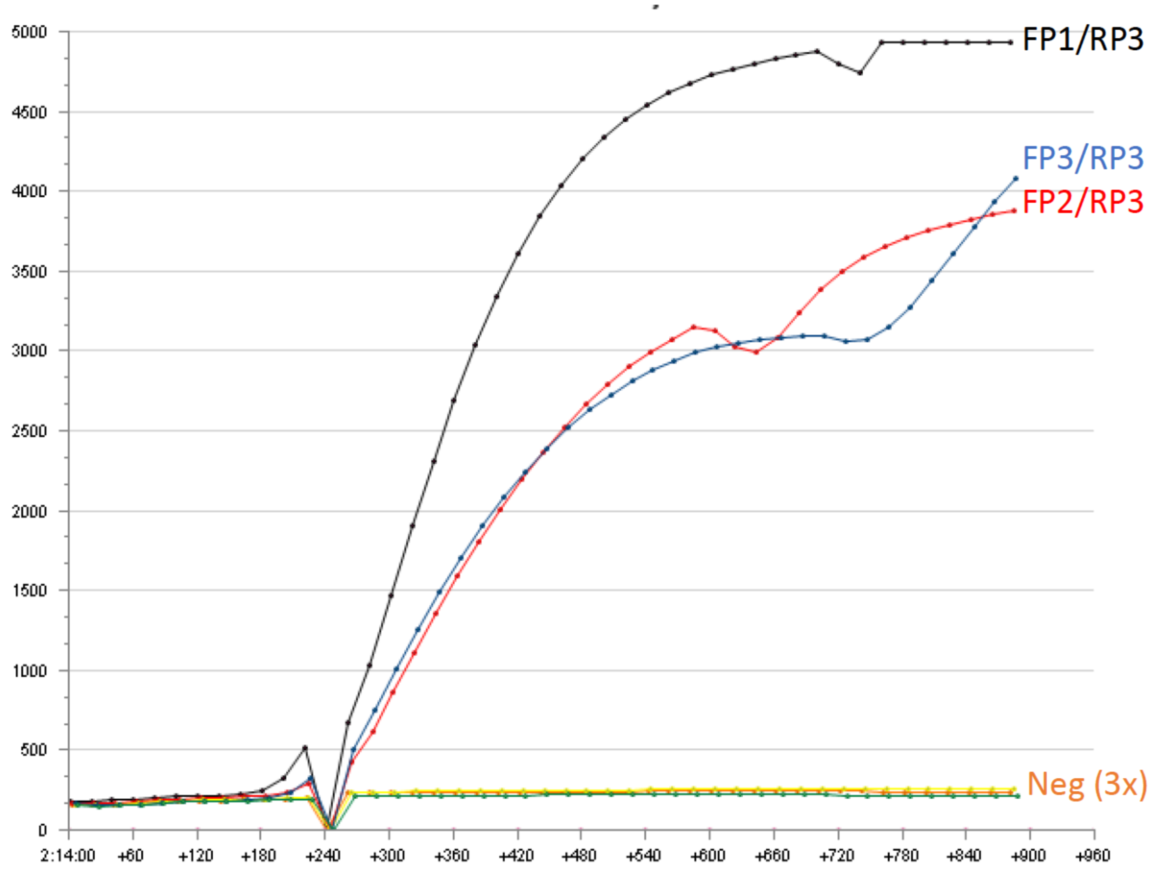

Figure S2. The three best primer combinations tested with 5*10^{5} ASFV molecular standard.

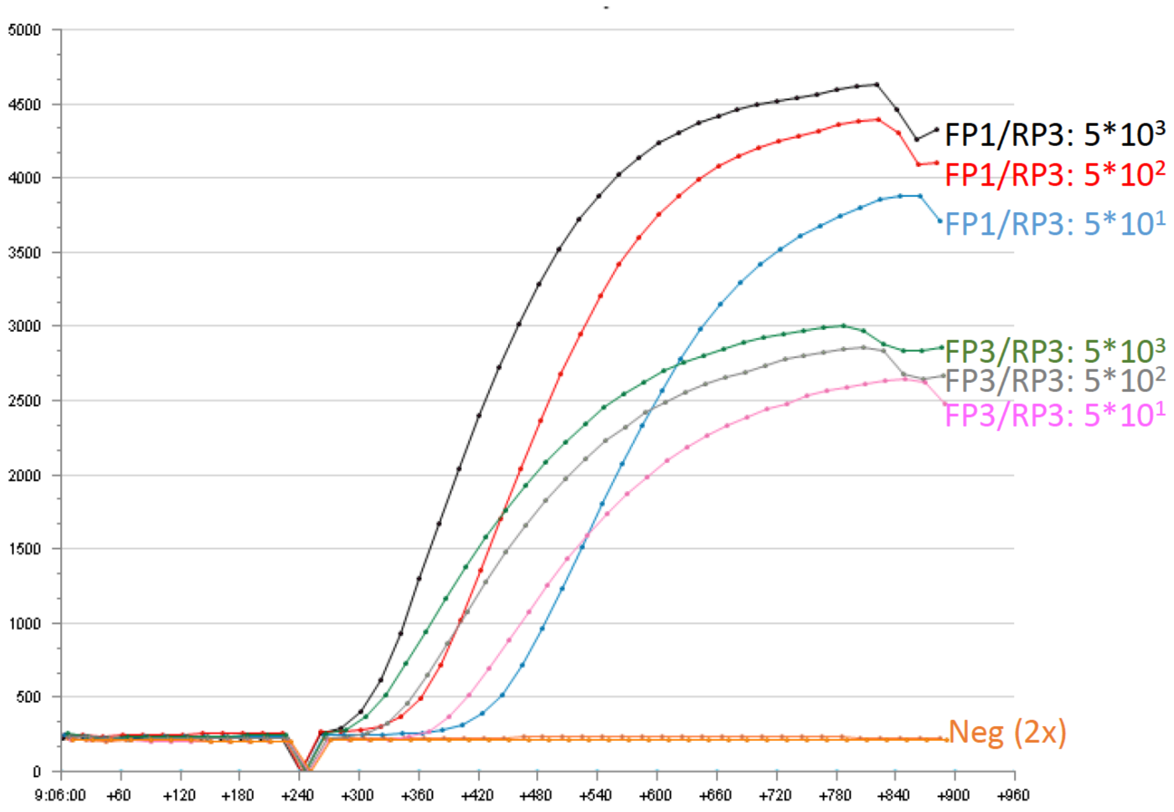

Figure S3. The two best primer combinations were tested with $5*10^{3-1}$ ASFV molecular standard. FP1/RP3 was confirmed to perform best.

Table S1. ASFV positive samples. ASFV Genotype and sample matrix were listed.

Sample ID	Matrix	Genotype	RPA	
			Result	TT (min)
1	Spleen	I	pos	2.35
2	Lung	I	pos	3.05
3	Macrophage culture	I	pos	3.01
4	Macrophage culture	I	pos	3.43
5	Spleen	II	pos	4.73
6	Lung	II	pos	4.76
7	Macrophage culture	II	pos	3.72
8	Blood	II	pos	4.73
9	Blood	II	pos	4.76
10	Blood	II	pos	3.35
11	Blood	II	pos	4.66
12	Blood	II	pos	3.06
13	Blood	II	pos	4.83
14	Blood	II	pos	4.76
15	Blood	II	pos	4.80
16	Blood	II	pos	4.81
17	Blood	II	pos	5.01
18	Blood	II	pos	3.05
19	Blood	II	pos	3.40

20	Blood	II	pos	3.43
21	Blood	II	pos	3.11
22	Blood	II	pos	3.48
23	Blood	II	pos	3.38
24	Blood	IV	pos	6.01
25	Blood	IV	pos	2.71
26	Blood	IV	pos	2.68
27	Blood	IV	pos	2.73
28	Blood	IV	pos	3.43
29	Spleen	IX	pos	3.12
30	Lung	IX	pos	3.48
31	Macrophage culture	IX	pos	3.73
32	Blood	XI	pos	4.76
33	Blood	XI	pos	4.78
34	Blood	XI	pos	4.81
35	Blood	XII	pos	4.45
36	Blood	XII	pos	4.48
37	Blood	XII	pos	5.01
38	Blood	XII	pos	4.71
39	Blood	XII	pos	3.40
40	Blood	XIII	pos	4.83
41	Blood	XIII	pos	4.86
42	Blood	XIII	pos	4.68
43	Blood	XIX	pos	4.76
44	Blood	XIX	pos	4.78
45	Blood	XIX	pos	4.81
46	Blood	XIX	pos	5.01
47	Blood	XIX	pos	5.05

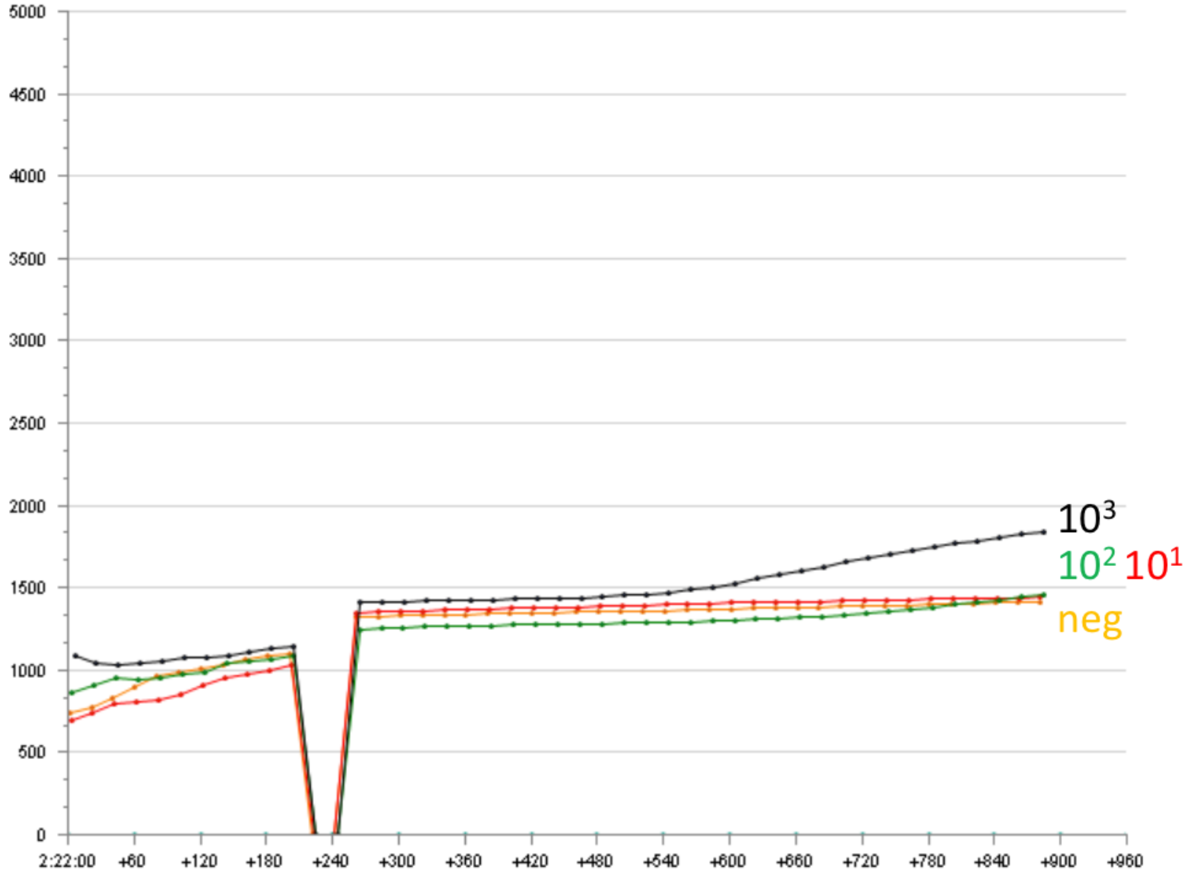

Figure S4. RPA assay using 10^{3-1} of the ASFV molecular standard based on Wang et al. 2020. Background was very high and only 10^3 showed a late weak signal.
